# Posterior parasagittal in-plane ultrasound-guided infraclavicular brachial plexus block–a case series

**DOI:** 10.1186/s12871-015-0090-0

**Published:** 2015-07-21

**Authors:** Zhi Yuen Beh, M. Shahnaz Hasan, Hou Yee Lai, Normadiah M. Kassim, Siti Rosmani Md Zin, Kin Fah Chin

**Affiliations:** 1 Department of Anaesthesiology, Faculty of Medicine, University of Malaya, 50603 Kuala Lumpur, Malaysia; 2 Department of Anatomy, Faculty of Medicine, University of Malaya, 50603 Kuala Lumpur, Malaysia; 3 M.I.L.E.S Training Centre, University of Malaya, 50603 Kuala Lumpur, Malaysia

## Abstract

**Background:**

The brachial plexus at the infraclavicular level runs deeper compared to its course proximally, giving rise to impaired needle visualisation due to the steep angle of needle insertion with the current ultrasound-guided approach. A new posterior parasagittal in-plane ultrasound-guided infraclavicular approach was introduced to improve needle visibility. However no further follow up study was done.

**Methods:**

We performed a case series and a cadaveric dissection to assess its feasibility in a single centre, University of Malaya Medical Centre, Kuala Lumpur, Malaysia from November 2012 to October 2013. After obtaining approval from the Medical Ethics Committee, University Malaya Medical Centre, 18 patients undergoing upper limb surgery were prospectively recruited. A cadaveric dissection was also performed. The endpoints of this study were the success rate, performance time, total anaesthesia-related time, quality of anaesthesia and any incidence of complications.

**Results:**

All patients had 100 % success rate. The imaging time, needling time and performance time were comparable with previously published study. There were no adverse events encountered in this study. The cadaveric dissection revealed a complete spread of methylene blue dye over the brachial plexus.

**Conclusion:**

This study demonstrated that the posterior parasagittal in-plane approach is a feasible and reliable technique with high success rate. Future studies shall compare this technique with the conventional lateral parasagittal in-plane approach.

**Trial registration:**

ClinicalTrials.gov NCT02312453. Registered on 8 December 2014.

## Background

Our study focus on the ultrasound guided infraclavicular brachial plexus block, which is a cord-level block of the brachial plexus for surgical procedures below mid-humerus. The brachial plexus at this level runs deeper compared to its course proximally, giving rise to impaired needle visualisation due to the steep angle of needle insertion with the current ultrasound-guided approach (lateral para-sagittal in-plane technique) [1]. A new ultrasound-guided posterior approach parasagittal in-plane infraclavicular block was introduced to improve needle visibility [2]. However no further follow up study was done.

Therefore, we performed a case series of 18 patients with a cadaveric dissection, to assess the feasibility of this approach.

## Methods

After obtaining ethics committee approval from the Medical Ethics Committee, University Malaya Medical Center, Kuala Lumpur, Malaysia (Chairperson Professor Dr. Looi Lai Meng; IRB reference no. 949.14 dated 17 October 2012, amendment no. 1038.76 dated 19 December 2013) and written informed consent, 18 patients undergoing surgery of the elbow, forearm, wrist, or hand were prospectively recruited based on the criteria below.

The inclusion criteria were patient’s age between 18 and 80 years old, American Society of Anesthesiologists (ASA) physical status I – III, body mass index (BMI) between 20 and 35 kg/m^2^ and planned for surgery of the forearm, wrist, or hand. The exclusion criteria were patient’s inability to give consent to the study, pre-existing neuropathy, infection at the site of puncture, coagulopathy, and allergy to amides local anaesthetics.

Prior to block, an intravenous cannula was inserted at the upper limb contralateral to the surgical site at the induction room. Premedication was given (intravenous midazolam 1–3 mg and/or fentanyl 25–50 ug) and supplemental oxygen via nasal cannulas at 3 L/min was administered. Standard ASA monitoring (non-invasive blood pressure, electrocardiogram, and pulse oximetry) was applied throughout the procedure.

All patients were given a single shot ultrasound-guided posterior parasagittal in-plane approach infraclavicular brachial plexus block under aseptic technique by one of the three operators (BZY, MSH and LHY). The blocks were performed using a 21G, 100 mm insulated short bevel needle (Stimuplex A, B Braun, Melsungen, Germany) without nerve stimulation. A 25-ml local anaesthetic admixture [Lignocaine 2 % (100 mg) plus Ropivacaine 0.75 % (150 mg)] was administered. We used an ultrasound machine (Sonosite M-Turbo; Sonosite®, Bothell, WA, USA) with HFL38x/ 13–6 MHz linear transducer probe.

Patient’s arm was allowed to rest in a neutral position by the side during the procedure. The infraclavicular area was cleaned with aqueous iodine solution and draped. The ultrasound probe was covered with sterile sheath and sterile gel applied.

**Fig. 1:**
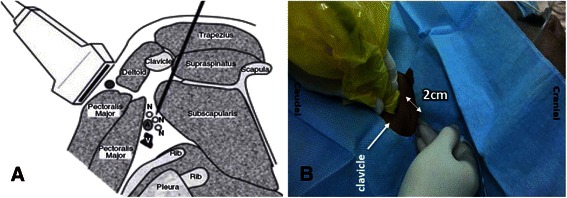
Ultrasound guided posterior approach to the infraclavicular brachial plexus. **a** Parasagittal section through the shoulder medial to the coracoid process showing block needle and ultrasound probe. (With permission from John Wiley and Sons; Ultrasound guided posterior approach to the infraclavicular brachial plexus. *Anaesthesia* 2007; **62**: 539). **b** Ideal needle insertion point – 2 cm posterior to clavicle to avoid needle contact with the inferior surface of the clavicle

The ultrasound probe was placed below the clavicle and medial to the coracoid process in the delto-pectoral groove i.e. para-sagittal view. A short-axis view of the axillary artery was obtained. We adopted the technique as described by Hebbard et al. [2]. A skin wheal was made with 3 mL lignocaine 1 %. The needle insertion point was over the trapezius muscle sufficiently posterior to allow the needle to pass between the clavicle and the scapula in the direction of the axillary artery. The insertion point was strictly aligned with the long axis of the ultrasound beam i.e. in-plane technique. During our pilot study, we identified the ideal needle insertion point would be 2 cm posterior to the clavicle to avoid needle tip contact with the inferior surface of the clavicle (Fig. 1).

The needle was advanced until a fascial click was felt when its tip reached the posterior aspect of the axillary artery (6 o’clock position) which indicated penetration of the septum posterolateral to the artery, confirming a good needle position with a high chance of block success [3, 4]. At this point, local anaesthetic was deposited incrementally each time after a negative aspiration, ensuring a U-shaped distribution of local anaesthetic with anterior displacement of the axillary artery, known as ‘double bubble sign’ [3, 4].

We adopted and modified the data collection and assessment method as described by Tran et al. [5–7]. The anaesthesia assistant recorded the imaging time (defined as the time interval between contact of the ultrasound probe with the patient and the acquisition of a satisfactory sonoanatomy – a complete round short-axis view of the axillary artery), needling time (defined as the time interval between the start of the needle insertion and the end of local anaesthetic injection through the needle) and performance time (defined as the sum of imaging and needling times). The incidence of paraesthesia and vascular puncture was recorded if any.

We assessed the adequacy of motor and sensory blockade at predetermined intervals, every 5 min until 30 min; time zero was defined as the time at which the block needle exited the skin. Sensory blockades of the musculocutaneous, median, radial, and ulnar nerves were graded according to a 3-point scale using a pin prick test, with relative comparison to pin prick sensation in the contra-lateral limb: 0 = no block, 1 = analgesia (patient could feel touch but not sharp), and 2 = anaesthesia (patient could not feel touch). The sites for sensory assessment were: musculocutaneous nerve – lateral aspect of the forearm, radial nerve – the lateral aspect of the dorsum of the hand, ulnar nerve – the volar aspect of the fifth finger, median nerve – the volar aspect of the thumb. Motor blockades were also graded on 3-point scale with relative comparison to the contra-lateral limb: 0 = no block, 1 = paresis, and 2 = paralysis. The motor function of each nerves were assessed according to its functional movement: musculocutaneous nerve – elbow flexion or forearm supination; radial nerve – thumb extension, wrist and fingers extension; ulnar nerve – thumb adduction or fingers adduction, abduction or flexion of little & ring finger; median nerve – thumb opposition or flexion of index & middle finger. The overall maximal composite score was 16 points. We considered patient was ready for surgery when a minimal composite score of 14 points was achieved, provided the sensory block score was equal or superior to 7 of 8 points. The onset time was defined as the time required to obtain 14 points. Therefore, the anaesthesia-related time was equal to the sum of the performance and onset time.

Following the 30-min block assessment, if the composite score was less than 14 points, a supplemental rescue forearm peripheral nerve blockade, local anaesthetic infiltration by surgeon, or conversion to general anaesthesia was employed at the discretion of the operating anaesthetist. For these patients, we did not record the onset time and classified them as failed block. Success rate was equivalent to surgical anesthesia, defined as the ability to proceed with surgery without the need for intravenous narcotics, general anaesthesia, rescue blocks or local infiltration by the surgeon [5–7]. If patient experienced anxiety as voiced by themselves or determined by the treating anaesthetist, additional administration of intravenous midazolam or propofol was given. Supplemental oxygen was administered during surgery.

The incidence of tourniquet pain, Horner’s syndrome, dyspnoea and symptoms suggestive of local anaesthetic toxicity were routinely checked. Postoperatively, patient was served with oral analgesic medication (such as paracetamol, non-steroidal anti-inflammatory drugs) at the justification of the surgeon and allergy history. A week after the surgery, all patients were contacted via phone by our acute pain service (APS) team to enquire about complications such as persistent paraesthesia or motor deficit.

We performed additional evaluation of the ultrasound guided posterior approach infraclavicular brachial plexus block on a cadaver. Similar methodology was employed with a total volume of normal saline 0.9 % 25 ml mixed with methylene blue (0.2 ml) was given. With the help of the anatomists, we dissected the right upper limb and evaluated the spread of the dye solution.

Statistical analysis was performed using SPSS version 20 statistical software (SPSS, IBM Corp). Continuous variables were presented as means (SDs); categorical variables were presented as counts or percentages.

## Results

We performed this study on 18 patients, 11 men and 8 women with a mean age of 37.7 years (SD 13.9 years) and mean body mass index of 26.6 kg/m^2^ (SD 4.1 kg/m^2^). In terms of ASA physical status, 11 patients were class I, 6 class II and 1 class III. 7 patients underwent hand surgery, 4 for wrist surgery, 6 for forearm surgery and 1 for elbow surgery (Table 1).

**Table 1:** Patient characteristics

Sex (male/female), n	11/7
Age, mean(SD), y	37.7(13.9)
BMI, mean(SD), kg/m^2^	26.6(4.1)
ASA physical status (I,II,III), n	11/6/1
Types of surgery (hand/wrist/forearm/elbow), n	7/4/6/1

Continuous variables were presented as means(SDs); SD, standard deviation; categorical variables were presented as counts. BMI indicates body mass index, ASA indicates American Society of Anesthesiologists

**Fig. 2:**
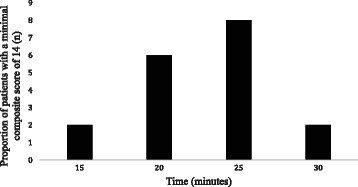
Proportion of patients with a minimal composite score of 14 points according to time. Most patients achieved readiness to undergo surgery (also defined as block onset time) by 25 min

We achieved 100 % success rate in all patients. None of these patients required the need for intraoperative intravenous narcotics, rescue blocks or local infiltration by the surgeon during operation and no conversion to general anaesthesia. The posterior technique seemed to have a fairly short imaging time (29 s [SD, 15 s]), needling time (4 min 31 s [SD, 1 min]), performance time (5 min 3 s [SD, 1 min 5 s]), onset time (22 min 46 s [SD, 4 min 16 s]) and total anaesthesia related time (27 min 50 s [SD, 4 min 36 s]) (Table 2). Most of them achieved composite score of 14 (readiness to undergo operation) by 25 min (Fig. 2).

**Table 2:** Block performance data

Imaging time, mean (SD), min: sec	0:29(0:15)
Needling time, mean (SD), min: sec	4:31(1:00)
Performance time, mean (SD), min: sec	5:03(1:05)
Onset time, mean (SD), min: sec	22:46(4:16)
Total anaesthesia related time, mean (SD), min: sec	27.50(4.36)
Success rate - surgical anaesthesia, n (%)	18(100.0)
Paraesthesia, n (%)	5(27.8)
Vascular puncture, n, (%)	0(0)
Tourniquet pain, n (%) / total cases required tourniquet application	0(0)/13

Continuous variables were presented as means (SDs); SD, standard deviation; categorical variables were presented as count or percentage. Imaging, needling, performance, and total anaesthesia-related times were calculated only for patients with a composite score of 14 points at 30 min

27.8 % of the patients reported incidence of paraesthesia during the procedure but follow up on all of them 1 week after surgery revealed no persistent paraesthesia or motor deficit. There were no adverse events occurred in this study. No incidence of vascular puncture and none experienced tourniquet pain (there were a total of 13 cases required tourniquet application during surgery) (Table 2). No Horner’s syndrome observed. No patient had dyspnoea or symptoms suggestive of LA toxicity.

**Fig. 3:**
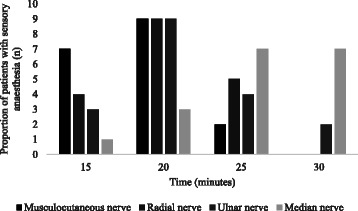
Proportion of patients with sensory anaesthesia (score of 2) according to time in the cutaneous distributions of nerves. The musculocutaneous nerve achieved fastest onset of sensory anaesthesia, followed by radial nerve. The ulnar and median nerve tend to be slower in achieving sensory anaesthesia

**Fig. 4:**
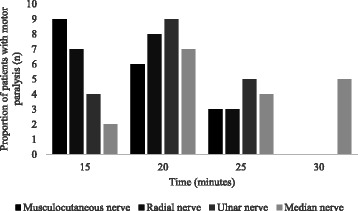
Proportion of patients with motor paralysis (score of 2) according to time in distributions of nerves. The musculocutaneous nerve achieved fastest onset of motor paralysis, followed by radial nerve. The ulnar was third and median nerve tend to be the slowest in achieving motor paralysis

From the Figs. 3 and 4, the posterior approach exhibited similar pattern of sensory and motor blocks profile. The musculocutaneous nerve was the fastest to achieve sensory anaesthesia and motor paralysis, followed by radial nerve, ulnar nerve and median nerve tend to be the slowest to achieve full blockade.

**Fig. 5:**
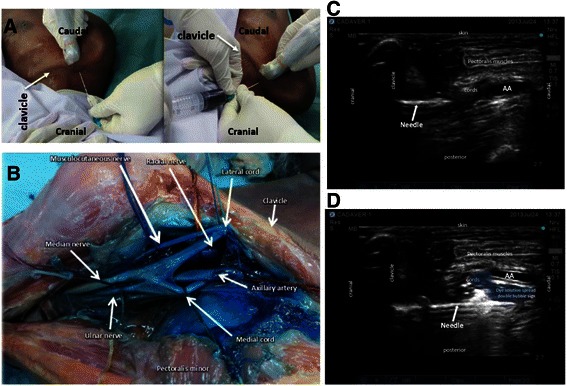
Cadaveric dissection: Ultrasound guided posterior parasagittal in-plane infraclavicular right brachial plexus block (**a**) needle insertion posterior to clavicle plus injection of dye solution 25 ml normal saline plus 0.2 ml methylene blue, (**b**) Right brachial plexus; Note the median and ulnar nerves were less stained compared to musculocutaneous and radial nerves (**c**) needle advancement after passing clavicle, needle trajectory - horizontal, easy direction towards target point, good needle visualization (**d**) Dye solution deposit on posterolateral aspect of axillary artery, creating double bubble sign

For the cadaveric dissection, we observed the distribution and spread of the methylene blue dye after the block. We could see that the median and ulnar nerves were less stained as compared with musculocutaneous and radial nerves (Fig. 5).

## Discussion

In this case series combined with a cadaveric dissection, we evaluated the feasibility of a single shot ultrasound guided posterior approach, parasagittal in-plane infraclavicular brachial plexus block. The results of this study showed that the posterior approach was a feasible technique with high success rate.

The posterior approach had comparable imaging, needling and performance times with conventional method based on previously published data. In a study conducted by Tran et al., 44 patients underwent operations with conventional approach ultrasound guided infraclavicular brachial plexus blocks [5]. The mean imaging time was 39 s (SD, 39 s), needling time was 4.5 min (SD, 1.4 min) and performance time was 5.1 min (SD, 1.5 min).

**Fig. 6:**
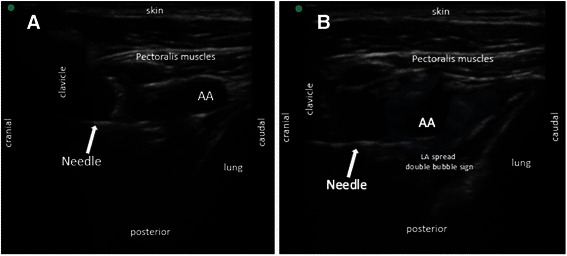
Ultrasound guided posterior parasagittal in-plane infraclavicular brachial plexus block (**a**) needle trajectory - horizontal, easy direction towards target point, good needle visualization in most cases (**b**) LA deposit on posterolateral aspect of axillary artery, creating double bubble sign

In the posterior approach, the needle would not be visible initially as it was obscured by the clavicle shadow. It would only appear on the ultrasound screen after it had travelled for some distance under the surface of the clavicle. As the needle trajectory was less acute compared to the conventional technique, it would appear in a horizontal fashion and almost directly perpendicular to the ultrasound beam. The needle therefore became more visible due to minimization of refraction and maximization of reflection of ultrasound beam towards the probe (Fig. 6). With deeper nerve targets, the angle of incidence between the structure and the ultrasound beam was more parallel resulting in more ultrasound waves being refracted and reflected away and fewer waves successfully return to the probe. Hence, the needle appeared less visible making the technique more challenging especially for novices.

**Fig. 7:**
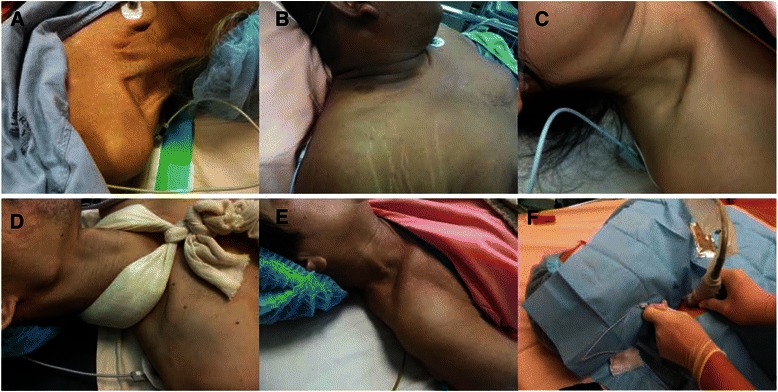
**a**–**e** Anatomical variations of the clavicles, (**f**) the needle head hit against patient’s head, limit the space for the operator to manipulate the needle

Despite having good needle visualization with the posterior approach, there were technical difficulties that we faced during the performance of this block. The factors that contributed to this were the size of the neck and its length, various anatomical variations of the clavicle and the size of the area over the supraclavicular fossa (Fig. 7). Short and thick neck would hinder and obstruct the pathway of needle insertion especially when the length of the needle used was quite long as in this case series (Fig. 7f). The shape of the clavicle and its various anatomical variations were also found to influence the size of the area above the clavicle. Clavicles which are more angulated in its lateral portion would reduce this area, hence contributing significantly to needling difficulty. From our case series, we found that the best position for this approach was to get the patient’s head to lie flat on the bed or trolley (without pillow) with the head turned to the contralateral side. A sandbag could also be placed underneath the shoulder to increase the space between neck and supraclavicular fossa.

5 or 27.8 % of the patients reported incidence of paraesthesia during the procedure but follow up on all of them 1 week after surgery revealed no persistent paraesthesia or neurological deficit. In a recent study of more than 7000 peripheral nerve and plexus blocks, 30 patients (0.5 %) were referred for neurological assessment [8]. Of these 30 patients, only three met the criteria for nerve injury related to peripheral nerve block (0.04 %). This study confirms that neurological deficits after peripheral nerve block are rare. However, neurological assessment and follow-up until resolution of the condition is vital.

The posterior approach showed similar pattern of sensory and motor blocks profile. The musculocutaneous nerve, being a branch from the lateral cord was the fastest to achieve sensory anaesthesia and motor paralysis. Likewise, radial nerve which branch from the posterior cord was the second fastest (almost as quick as musculocutaneous nerve) to achieve full blockade. Sauter et al. observed that, in most of subjects, the lateral cord lies approximately at 9-o’ clock, 276° (263°–321°) and posterior cord lied at 8-o’ clock, 236° (189°–261°) from the center of the artery [9]. Rapid blockade of both nerves were due to their close proximity to the target point site of local anaesthetic injection. The ulnar and median nerves are both branches of medial cord, though median nerve also received contribution from the lateral cord. These two nerves tend to take longer time to achieve full blockade. The medial cord usually lies on the medial aspect of the axillary artery, at 159° (90°–290°) from the center of the artery making local anaesthetic spread to the structure the slowest to take effect [9].

As for the cadaveric dissection, we observed the distribution and spread of methylene blue dye after performing the block. The imaging and needle visibility were excellent because this cadaver was thin in size. We could see that the median and ulnar nerves were less stained as compared with musculocutaneous and radial nerves (Fig. 5), which correlates with the findings of the timing of block onset of each nerve in our study.

The limitation of this study was its small sample size (18 patients and 1 cadaver specimen). The main difference between this block approach and the conventional infraclavicular approach is the site and angle of needle insertion. Otherwise, the end point of local anaesthetic injection remained the same for both approaches. We stopped recruiting after performing the blocks in these 18 patients because all the blocks had a hundred percent success rate and we did not encounter any major complications other than the technical difficulties as described above. We felt that the number of subjects was adequate and further evaluation of this approach shall be a randomised trial comparing it with the conventional technique. Another limitation of this study is the lack of description with regard to the clarity of the visualised needle.

## Conclusion

This study demonstrated that the posterior parasagittal in-plane approach is a feasible and reliable technique with high success rate. Future studies shall compare this technique with the conventional lateral parasagittal in-plane approach.
